# Multi-phase-combined CECT radiomics models for Fuhrman grade prediction of clear cell renal cell carcinoma

**DOI:** 10.3389/fonc.2023.1167328

**Published:** 2023-08-23

**Authors:** Zhiyong Zhou, Xusheng Qian, Jisu Hu, Chen Geng, Yongsheng Zhang, Xin Dou, Tuanjie Che, Jianbing Zhu, Yakang Dai

**Affiliations:** ^1^ Suzhou Institute of Biomedical Engineering and Technology, Chinese Academy of Sciences, Suzhou, Jiangsu, China; ^2^ School of Biomedical Engineering (Suzhou), Division of Life Sciences and Medicine, University of Science and Technology of China, Suzhou, Jiangsu, China; ^3^ Department of Pathology, Second Affiliated Hospital of Soochow University, Suzhou, Jiangsu, China; ^4^ Department of Radiology, Second Affiliated Hospital of Soochow University, Suzhou, Jiangsu, China; ^5^ Key Laboratory of Functional Genomic and Molecular Diagnosis of Gansu Province, Lanzhou, Gansu, China; ^6^ Suzhou Science & Technology Town Hospital, Gusu School, Nanjing Medical University, Suzhou, Jiangsu, China

**Keywords:** clear cell renal cell carcinoma, radiomics, Fuhrman grading, multi-phase feature, texture

## Abstract

**Objective:**

This study aimed to evaluate the effectiveness of multi-phase-combined contrast-enhanced CT (CECT) radiomics methods for noninvasive Fuhrman grade prediction of clear cell renal cell carcinoma (ccRCC).

**Methods:**

A total of 187 patients with four-phase CECT images were retrospectively enrolled and then were categorized into training cohort (n=126) and testing cohort (n=61). All patients were confirmed as ccRCC by histopathological reports. A total of 110 3D classical radiomics features were extracted from each phase of CECT for individual ccRCC lesion, and contrast-enhanced variation features were also calculated as derived radiomics features. These features were concatenated together, and redundant features were removed by Pearson correlation analysis. The discriminative features were selected by minimum redundancy maximum relevance method (mRMR) and then input into a C-support vector classifier to build multi-phase-combined CECT radiomics models. The prediction performance was evaluated by the area under the curve (AUC) of receiver operating characteristic (ROC).

**Results:**

The multi-phase-combined CECT radiomics model showed the best prediction performance (AUC=0.777) than the single-phase CECT radiomics model (AUC=0.711) in the testing cohort (*p* value=0.039).

**Conclusion:**

The multi-phase-combined CECT radiomics model is a potential effective way to noninvasively predict Fuhrman grade of ccRCC. The concatenation of first-order features and texture features extracted from corticomedullary phase and nephrographic phase are discriminative feature representations.

## 1 Introduction

Renal cell carcinoma (RCC) is the seventh most common malignant tumors in humans, and its incidence is increasing 2% annually around the world (1). RCC is generally categorized into clear cell renal cell carcinoma (ccRCC) and non-clear cell renal cell carcinoma (non-ccRCC) (2). ccRCC is the most common histological subtype of RCC, accounting for more than 70% of all RCC cases (3). Compared with non-ccRCC, ccRCC receives more attentions in clinical practice due to its higher metastatic potential and worse prognosis (4).

The Fuhrman nuclear grade is a widely used grading system in the pathological nuclear grading of ccRCC (5), which is established as an independent histological prognostic factor and is significant for the clinical management of ccRCC (6). Fuhrman nuclear grade stratifies ccRCC tumors into four grades based on nuclear morphology (7). A preoperative percutaneous biopsy of renal masses is a widely used method and considered as a gold standard for histology diagnosis and treatment plan. However, it cannot roundly reflect the Fuhrman grades of the entire tumor and may be discordant with surgical histopathology (accuracy=46–64%) (8, 9) due to the high spatial heterogeneity and inherent genetic heterogeneity of ccRCC (10). Since more than 70% of renal masses are discovered incidentally on routine clinical imaging, preoperative noninvasive assessment of Fuhrman grade by using multi-phase contrast-enhanced CT (multi-phase CECT) has received widespread attentions (7).

In recent years, artificial intelligence methods using radiomics analysis have gradually attracted increased attention due to its excellent performance on cancer classification and survival prediction in radiation oncology. Radiomics methods translate medical imaging data into high-dimension features, which represent microscale information of tumors, e.g., tumor microenvironment, micro-vessel density, and irregularity of nuclear shape and arrangement. Theoretically, radiomics analysis is a machine learning architecture, which provides interpretable image features as noninvasive radiology biomarkers for auxiliary diagnose or prognosis (11–14). Some studies have shown that radiomics methods could provide valuable information for predicting benign and malignant tumors, tumor subtypes, and tumor grade for RCC (15–17). However, these previous studies are confined by the limitations that radiomics features were extracted from single-phase CECT images (usually extracted from nephrographic phase of CECT) (18, 19), which may lead to the loss of some tumor biological information (20), e.g., wash-in-and-wash-out.

The primary purpose of this study was to investigate the effectiveness and ability of multi-phase-combined CECT radiomics models with incorporated 3D classical radiomics and contrast-enhanced variation features to noninvasively distinguish low and high grades in simplified Fuhrman grading system of ccRCC.

## 2 Materials and methods

### 2.1 Patients

The study was approved by our local institutional review board. We collected CT images, histopathology reports, and clinical data of patients who had undergone surgical resections of ccRCC between January 2009 and January 2019.

A patient was included in this study if he/she underwent a preoperative CECT with a four-phase renal mass CT imaging protocol (unenhanced phase, corticomedullary phase, nephrographic phase, and delay phase) and had a histopathology report proven as ccRCC with a diagnosis of Fuhrman grades. The exclusion criteria were as follows: 1) lack of Fuhrman grades in histopathology reports (n=36); 2) lack of CT images (n=4); 3) incomplete contrast-enhanced phases (n=17); 4) incomplete lesion in CT images (n=2); and 5) suboptimal CT imaging quality (n=1).

**Figure 1:**
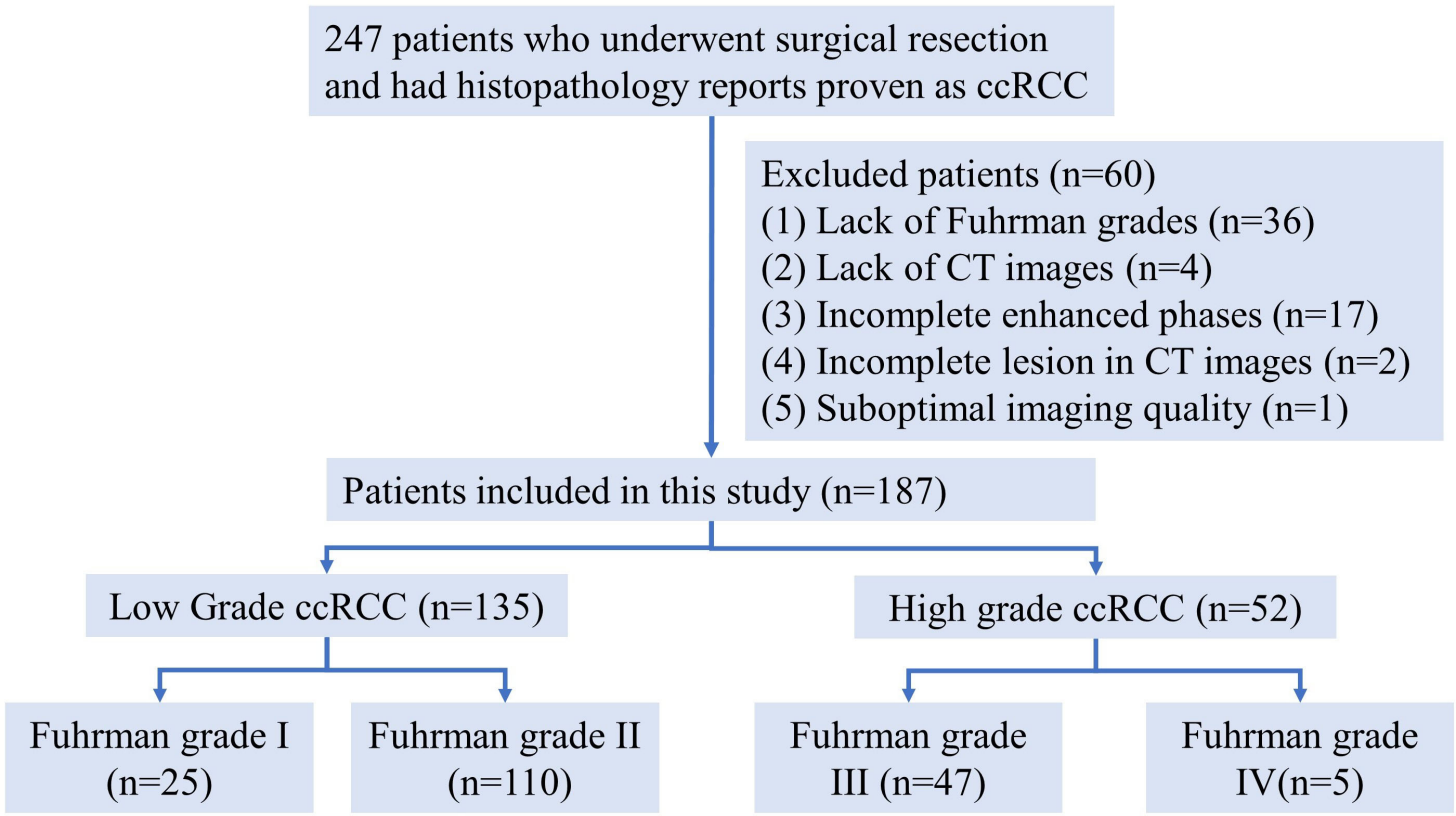
Patient recruitment flowchart.

A total of 187 samples were finally enrolled in our retrospective study. All samples were categorized into low- and high grade according to the simplified Fuhrman grade system. **Figure 1** portrays the patient recruitment flowchart.

### 2.2 Fuhrman stage

In order to ensure reproducibility of pathological diagnosis and reduce the intra-/inter-observer variability (5), the traditional four-tiered Fuhrman grading system (FGS) was re-categorized into a simplified Fuhrman grading system with low grade (corresponding to grade I and II in the traditional FGS) and high grade (grade III and IV in the traditional FGS). The simplified FGS could predict prognosis and cancer-specific mortality as good as the traditional four-tiered FGS, which is also widely used in clinical practice (21). Fuhrman grading was accomplished by a genitourinary pathologist with 12 years of experience.

### 2.3 Four-phase renal mass CT imaging protocol

Preoperative four-phase CECT images were acquired from our institutional PACS. All patients underwent CT on a 64-channel MD-CT scanner (GE, Philips). The acquisition parameters were as follows: tube voltage, 120 kV; auto tube current, 200–400 mA depended on patient size; reconstruction slice thickness, 0.625–1.5 mm; pitch, 0.984; collimation, 0.625×64 mm; and tube rotation of 0.5 s. The contrast agent for enhanced scanning is iohexol injection (300 mgI/ml, Yangzi River Pharmaceutical Co., Ltd., Taizhou, Jiangsu Province, China), the dosage of contrast agent is 0.5gI/kg body weight, and the injection time of contrast agent is fixed at 30 s.

All patients in this study underwent preoperative four-phase CECT scans: unenhanced phase, corticomedullary phase, nephrographic phase, and delay phase. The timing of the corticomedullary phase was established by bolus tracking (GE Medical Systems, Beijing, China), which was used to determine the onset of imaging. A circular region of interest (ROI) was placed in the thoracoabdominal aorta junction with a trigger set to begin at 150 HU. CECT images were acquired at 10s (corticomedullary phase), 70–80s (nephrographic phase), and 120–180s (delay phase) after the threshold of 150 HU was reached.

### 2.4 Image preparing

**Figure 2:**
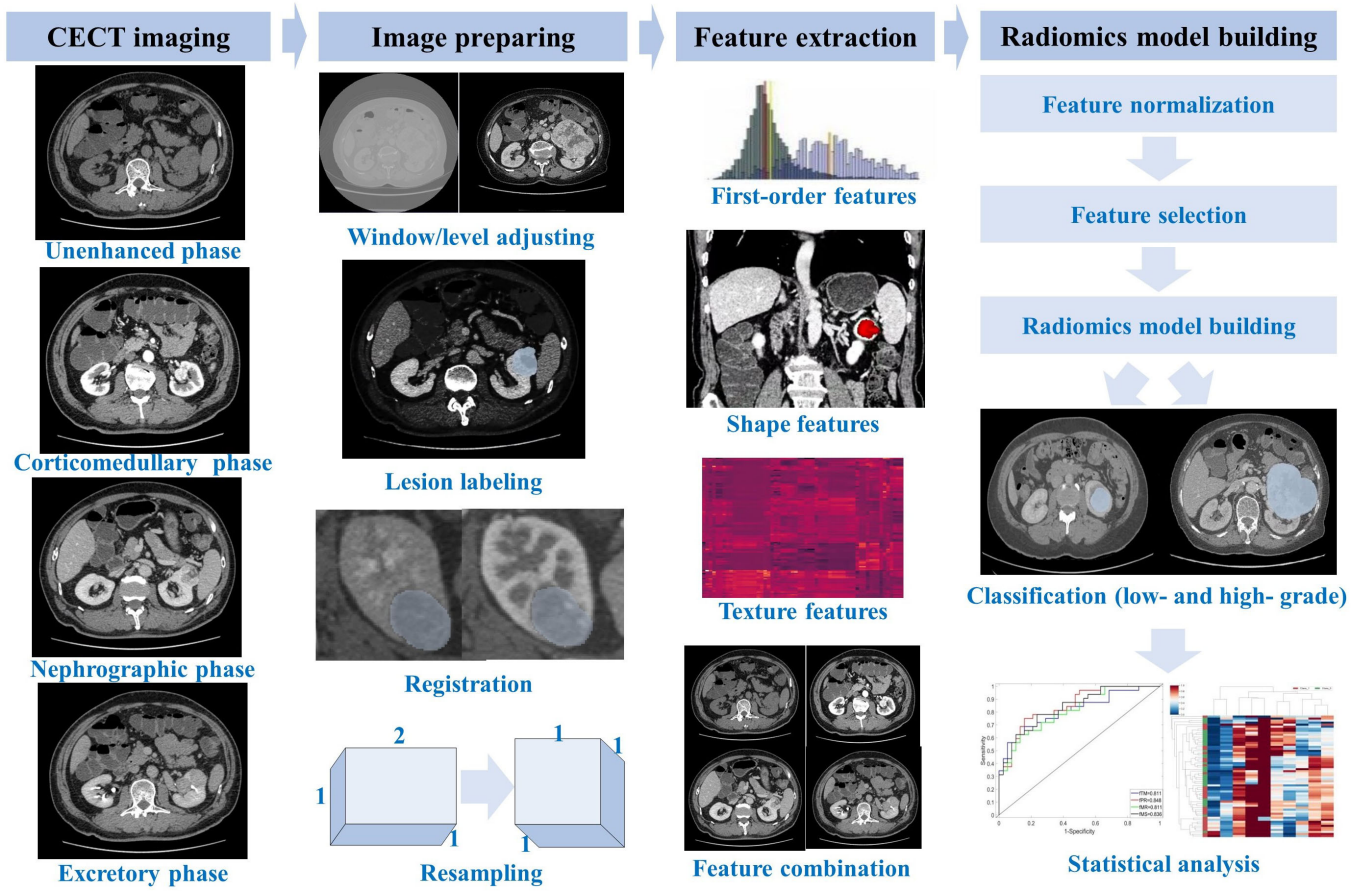
Flowchart of multi-phase-combined radiomics model building for Fuhrman grade prediction of ccRCC.

**Figure 2** shows the flowchart of building a multi-phase-combined CECT radiomics model for the Fuhrman grade prediction of ccRCC, which mainly includes three steps: image preparing, feature extraction, and radiomics model building. The workflow detail of building multi-phase-combined CECT radiomics model was described in the following subsections.

**Figure 3:**
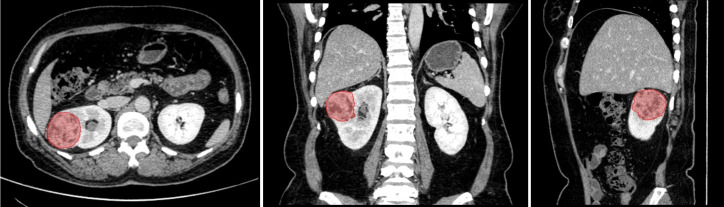
The segmentation of entire tumor mass (red region) on nephrographic phase CT. From left to right are axial, coronal and sagittal.

All CT images with anonymized DICOM format were retrieved from PACS. In order to obtain the target ROI in CECT images, two senior radiologists segmented the entire tumor masses (shown in **Figure 3**) by delineating the outline of all contiguous slices of the tumors in nephrographic phase CECT with itk-SNAP (http://www.itksnap.org/). Both the two radiologists were blinded to the segmentation of each other and to the clinical and histopathological reports. The ROIs of tumor masses in nephrographic phase CECT images were then applied to the other three phases with slight adjustments tailoring VOIs in each phase by an affine-registration method. The affine registration was performed by elastix (22) (https://elastix.lumc.nl/) to guarantee the corresponding voxels on different phases fits to each other.

Some other preprocessing methods were performed before radiomics feature extraction: all images were resampled into voxel spacing of 1 mm ×1 mm ×1 mm using B- spline interpolation, the size of bin width is 25, and the density value of each voxel was shifted 1,000 HU to guarantee a positive value.

### 2.5 Feature extraction

A total of 110 3D classical radiomics features were extracted from each ROI in single-phase CT image by using PyRadiomics (14), which is an open-source python package for radiomics feature extraction from medical images. These classical radiomics features could be categorized into seven types: first-order features (n=19), shape features (n=16), gray level co-occurence matrix features (GLCM, n=24), gray level run length matrix features (GLCM, n=16), gray level size zone matrix features (GLSZM, n=16), neighboring gray tone difference matrix features (NGTDM, n=5), and gray level dependence matrix features (GLDM, n=14). GLCM, GLCM, GLSZM, NGTDM, and GLDM are widely known as texture features. The detail of extracted feature names is listed in **Appendix A**. All features are in compliance with the definitions of Imaging Biomarker Standardization Initiative (23). In this study, the classical radiomics features were termed as $F_{1}^{pha}$ , $F_{2}^{pha}$ , $F_{3}^{pha}$ , and $F_{4}^{pha}$ corresponding to the unenhanced phase, corticomedullary phase, nephrographic phase, and delay phase. In order to comprehensively reflect the tumor biological information, features extracted from different phases were furtherly concatenated together to build multi-phase-combined radiomics models. Concretely, the concatenation of features combined with two phases were termed as $F_{1,2}^{pha}$ , $F_{1,3}^{pha}$ , $F_{1,4}^{pha}$ , $F_{2,3}^{pha}$ , $F_{2,4}^{pha}$ , and $F_{3,4}^{pha}$ , including 220 features in the two-phase feature set; the concatenation of features combined with three phases were termed as $F_{1,2,3}^{pha}$ , $F_{1,2,4}^{pha}$ , $F_{1,3,4}^{pha}$ , and $F_{2,3,4}^{pha}$ , including 330 features in the three-phase feature set; the concatenation of features combined with four phases were termed as $F_{1,2,3,4}^{pha}$ , including with 440 features in it.

**Figure 4:**
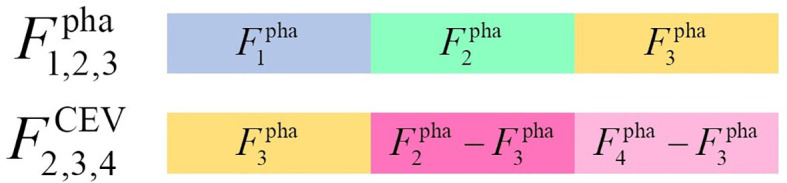
The samples of multiple-phase feature concatenation.

Variations in contrast enhanced between different phases were quantitative descriptors to represent wash-in-and-wash-out, which macroscopically reflected the hemodynamics and micro-vessel density of individual ccRCC lesion. However, the direct concatenation of classical radiomics features may be limited, since it is a challenge for common machine learning classifiers to mine the latent variations in contrast enhanced between different phases. Therefore, contrast-enhanced variation (CEV) features *F* ^CEV^ were proposed as


$$F_{i}^{CEV}=F_{i}^{pha}-F_{3}^{pha},\;i=1, 2, 4$$

of which $F_{i}^{pha}$ denoted the extracted feature from non-nephrographic phase and $F_{3}^{pha}$ was a corresponding feature extracted from the nephrographic phase. *F* ^CEV^ quantitatively represented the variation of first-order features and texture features between nephrographic phase and others phases. The nephrographic phase was selected as a fiducial phase in this study due to its key role in noninvasive auxiliary diagnose for ccRCC (24–27), although any phase could be theoretically considered as a fiducial phase to calculate CEV features. The concatenation of nephrographic phase features *F*_3_ and other single-phase CEV features were termed as $F_{1,3}^{CEV}$ (concatenation of $F_{3}^{pha}$ and $F_{1}^{pha}$ – $F_{3}^{pha}$ ), $F_{2,3}^{CEV}$ (concatenation of $F_{3}^{pha}$ and $F_{2}^{pha}$ – $F_{3}^{pha}$ ), and $F_{3,4}^{CEV}$ (concatenation of $F_{3}^{pha}$ and $F_{4}^{pha}$ − $F_{3}^{pha}$ ). The concatenation of nephrographic phase features $F_{3}^{pha}$ and two-phase CEV features were termed as $F_{1,2,3}^{CEV}$ (concatenation of $F_{3}^{pha}$ , $F_{1}^{pha}$ – $F_{3}^{pha}$ , and $F_{2}^{pha}$ – $F_{3}^{pha}$ ), $F_{1,3,4}^{CEV}$ (concatenation of $F_{3}^{pha}$ , $F_{1}^{pha}$ – $F_{3}^{pha}$ , and $F_{4}^{pha}$ – $F_{3}^{pha}$ ), $F_{2,3,4}^{CEV}$ (concatenation of $F_{3}^{pha}$ , *F*pha 2– $F_{3}^{pha}$ , and $F_{4}^{pha}$ – $F_{3}^{pha}$ ). The concatenation of nephrographic phase features and other three-phase CEV features were termed as $F_{1,2,3,4}^{CEV}$ (concatenation of $F_{3}^{pha}$ , $F_{1}^{pha}$ - $F_{3}^{pha}$ , $F_{2}^{pha}$ – $F_{3}^{pha}$ , and $F_{4}^{pha}$ – $F_{3}^{pha}$ ). Two examples ( $F_{2,3,4}^{pha}$ , $F_{2,3,4}^{CEV}$ ) of multiple-phase feature concatenation are shown as **Figure 4**.

A total of 22 types of image feature sets were finally constructed, which could be categorized into five groups: (1) single-phase feature group, including $F_{1}^{pha}$ , $F_{2}^{pha}$ , $F_{3}^{pha}$ , and $F_{4}^{pha}$ ; (2) two-phase-combined feature group, including $F_{1,2}^{pha}$ , $F_{1,3}^{pha}$ , $F_{1,4}^{pha}$ , $F_{2,3}^{pha}$ , $F_{2,4}^{pha}$ , and $F_{3,4}^{pha}$ ; (3) three-phase-combined feature group, including $F_{1,2,3}^{pha}$ , $F_{1,2,4}^{pha}$ , $F_{1,3,4}^{pha}$ , and $F_{2,3,4}^{pha}$ ; (4) four-phase-combined feature group: $F_{1,2,3,4}^{pha}$ ; and (5) CEV feature group, including $F_{1,3}^{CEV}$ , $F_{2,3}^{CEV}$ , $F_{3,4}^{CEV}$ , $F_{1,2,3}^{CEV}$ , $F_{1,3,4}^{CEV}$ , $F_{2,3,4}^{CEV}$ , and $F_{1,2,3,4}^{CEV}$ .

This study aimed to comprehensively investigate the effectiveness of multi-phase- combined radiomic features. Some existing methods were included in the 22 models. The single-phase models ( $F_{1}^{pha}$ , $F_{2}^{pha}$ , $F_{3}^{pha}$ , and $F_{4}^{pha}$ ) were equivalent to the method in (28), $F_{1,3}^{CEV}$ was equivalent to the method in (29), and $F_{1,2,3}^{pha}$ is equivalent to the method in (30).

### 2.6 Multi-phase-combined CECT radiomics model building

#### 2.6.1 Feature selection

As a preprocessing procedure of feature selection, each feature was standardized to achieve a zero mean and unit variance in the training cohort to avoid the effect of different scales. The feature of test cohort was standardized by applying the standardized hyperparameters (mean and standard deviation) obtained from the training cohort. In order to select discriminative features, a sophisticated feature selection procedure was performed as follows. First, low reproducibility features were removed if variance in normalized feature value was smaller than 10^−3^. Some uncertainty could be introduced into the tumor delineation. The segmentation of intra- and inter-observer was confirmed by two experienced radiologists. The intraclass correlation coefficients (ICCs) were computed by a two-way mixed-effect model to assess the inter- and intra-observer reproducibility. The features with ICC lower than 0.75 were considered as the poor agreement of the feature and therefore were removed. Second, Pearson correlation analysis (31) was performed to identify the distinctiveness of features and remove the redundant features if their absolute correlations were higher than 0.5. Some studies have shown that an additional feature ranking procedure was an effective way to improve the final performance of classification (32). Therefore, a multivariate ranking method, named as minimum redundancy maximum relevance (mRMR), was applied to identify the most important features on the criterion of both minimum redundancy and maximum relevance. Finally, only the top 20 important features in each features set were selected and input into a C-SVC (C-support vector classifier) (33) to build multi-phase-combined radiomics models. C-SVC is a widely used classifier based on support vector machine, which showed its efficiency and robustness in disease prediction (34–36).

#### 2.6.2 Modeling and statistical analysis

A total of 22 radiomics models were built to evaluate the ability and effectiveness of the proposed multi-phase-combined radiomics models. A total of 126 samples who underwent surgery before December 2016 were assigned into the training cohort, and the remaining 61 samples were assigned into the test cohort. We trained models and fine-tuned hyper-parameters by fivefold cross-validation with five repeated experiments in the training cohort to reduce the data selection bias and alleviate the overfitting. In the training cohort, the training data and validation data were randomly re-selected for fivefold cross-validation in each experiment. Finally, the model’s performance was assessed on the testing cohort, which was independent to the training cohort. The fine-tuned hyper-parameters were C=0.5, kernel=ploy, degree=7, and gamma=1/(feature number). The test cohort was used to assess the performance of all models by the area under the curve (AUC) of receiver operating characteristic curve (ROC). The statistical analysis was completed by using Python v3.8.

## 3 Results

### 3.1 Demographics

There were 187 patients (mean age, 58.80 ± 13.88 years; age range, 21–88 years) enrolled in this study, including 127 men (mean age, 59.76 ± 13.45 years; age range, 24–88 years) and 60 women (mean age, 56.77 years ± 13.63; age range, 21–83 years). There were 135 low-grade ccRCC patients (Fuhrman I: n=24, 12.83%; Fuhrman II: n=111, 59.36%) and 52 high-grade ccRCC patients (Fuhrman III: n=47, 25.13%; Fuhrman IV: n=5, 2.67%). The demographics and characteristics of the whole patient cohort are provided in **Table 1**. There were significant differences in gender between low grade and high grade (*p*<0.05), while there was no significant difference in age and tumor size between low grade and high grade (*p* > 0.05).

**Table 1:** Demographics and characteristics of the study population.

Characteristic	Total number n=187	Low grade (Fuhrman grade I and II) n=135	High grade (Fuhrman grade III and IV) n=52	*p*-value
Gender				
Male (n/%)	127 (67.91%)	88 (65.19%)	39 (75.00%)	<0.01^*a^
Female (n/%)	60 (32.09%)	47 (34.81%)	13 (25.00%)	
Age (mean ± STD, year)	58.80 ± 13.88	58.17 ± 14.27	60.50 ± 11.42	0.426^b^
Tumor size (mean ± STD, mm)	60.18 ± 30.43	57.21 ± 28.38	67.87 ± 35.17	0.095^b^

* p-value<0.05 was considered as statistically significant difference.

a Chi-square test.

b Independent t-test.

Specifically, 126 samples (low grade=93, high grade=33) who underwent surgery before December 2016 were assigned into the training cohort, and the rest 61 samples (low grade=42, high grade=19) were assigned into the test cohort. The demographics and characteristics of training and test cohorts are demonstrated in **Table 2**.

**Table 2:** Demographics and characteristics of the training cohort and validation cohort.

Characteristic	Training cohort			Validation cohort		
	Low grade	High grade	*p*-value	Low grade	High grade	*p*-value
Gender						
Male (n/%)	62 (65.26%)	29 (76.32%)	<0.01^*a^	26 (65.00%)	12 (75.00%)	<0.01^*a^
Female (n/%)	33 (34.74%)	9 (23.68%)		14 (35.00%)	4 (25.00%)	
Age (mean ± STD, year)	57.48 ± 15.67	63.19 ± 10.26	0.046 ^*b^	59.70 ± 10.04	54.44 ± 11.56	0.102 ^b^
Tumor size (mean ± STD, mm)	58.96 ± 27.58	60.15 ± 26.41	0.830^b^	53.37 ± 28.03	72.72 ± 39.33	0.071^b^

* p-value<0.05 was considered as statistically significant difference.

a Chi-square test.

b Independent t-test.

### 3.2 Performance of multi-phase-combined radiomics methods

#### 3.2.1 Discriminative capability

**Figure 5:**
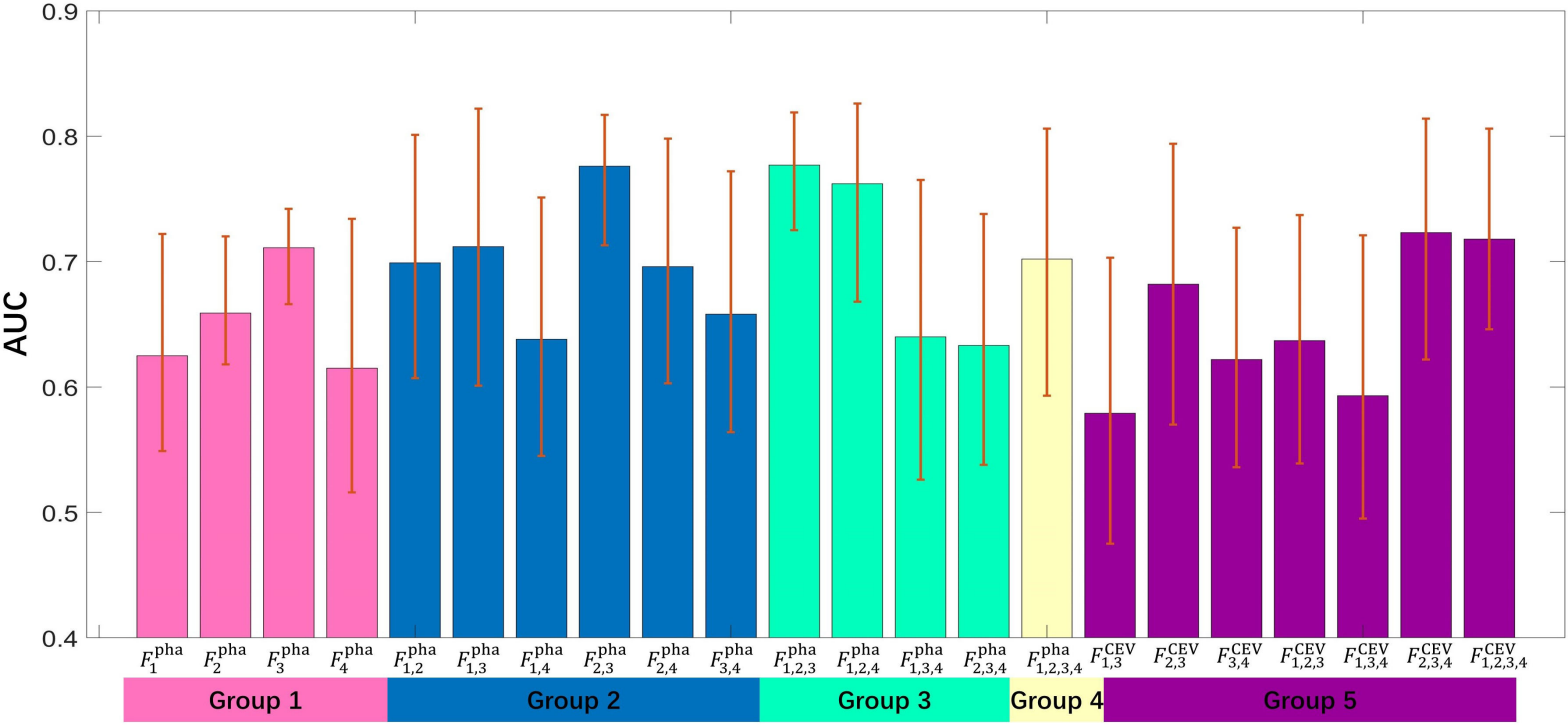
The bar height represents an AUC value of individual model; the error bar represents the upper and lower bounds of 95% CI.

**Figure 6:**
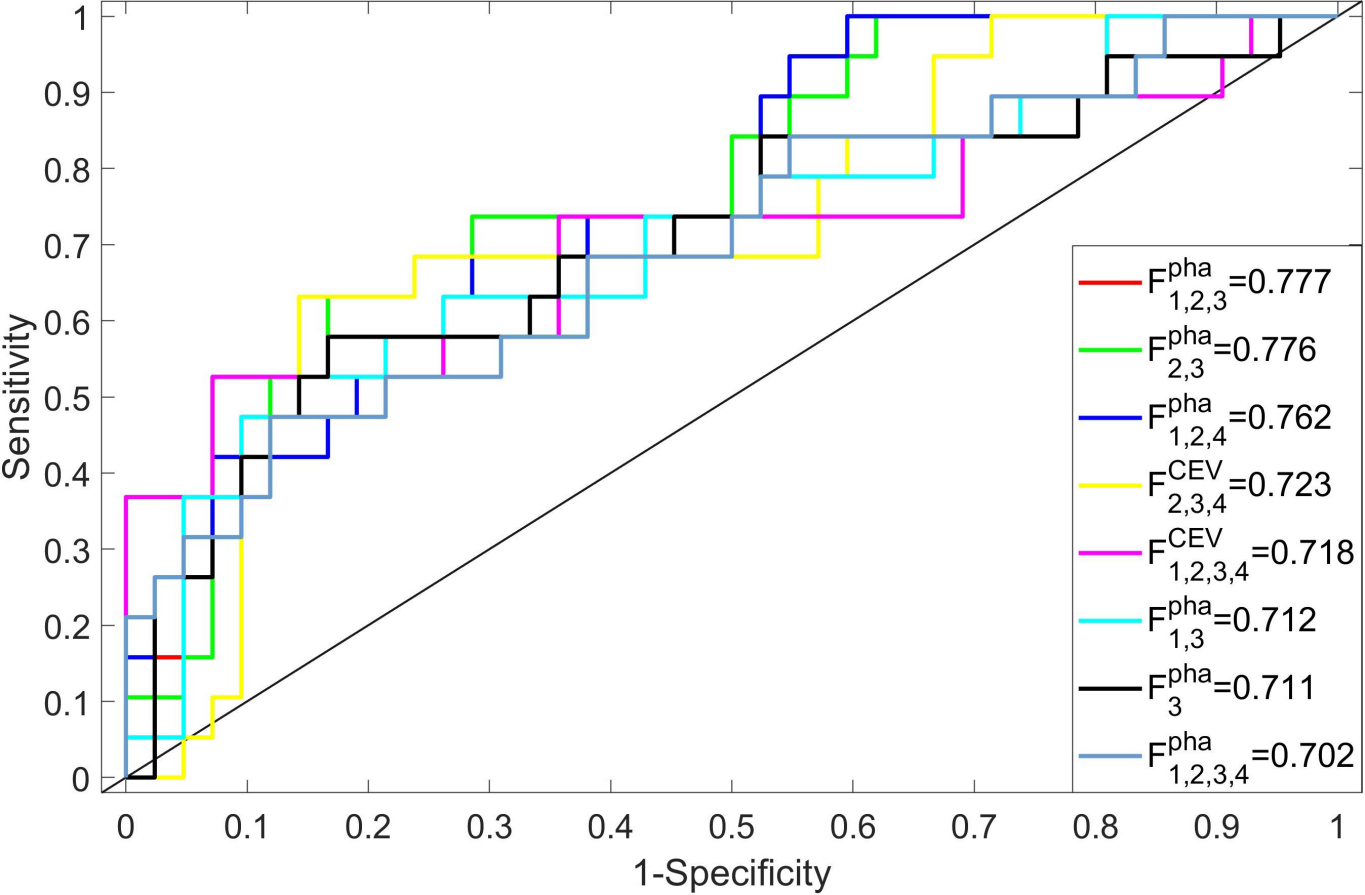
The performance (ROC and AUC value) of the discriminative models (AUC>0.7).

The 22 radiomics models were built by importing top 20 important features into a C-SVC classifier, and the optimized parameter configurations were determined by fivefold cross-validation with five repeated experiments. The training and validation data were randomly re-selected in each experiment. The discriminative capabilities of all radiomics models were evaluated on the test cohort with AUC, which are illustrated in **Figure 5**. The ROC value of an individual model was higher than 0.7 considered as a discriminative model in this study (26). Eight models showed their excellent discriminative capabilities: one model was built by single-phase features ( $F_{3}^{pha}$ ), five models were built by the concatenation of multi-phase features ( $F_{1,3}^{pha}$ , $F_{2,3}^{pha}$ , $F_{1,2,3}^{pha}$ , $F_{1,2,4}^{pha}$ , and $F_{1,2,3,4}^{pha}$ ), and two models were built by concatenation of CEV features ( $F_{2,3,4}^{CEV}$ and $F_{1,2,3,4}^{CEV}$ ). The ROC curves of these discriminative models are shown in **Figure 6**.

In comparison to other single-phase models, $F_{3}^{pha}$ showed its best performance (AUC=0.711), which suggested that features extracted from nephrographic phase may be considered as potential radiology biomarkers for ccRCC Fuhrman grade prediction. **Figure 6** also shows that six multi-phase-combined radiomics models ( $F_{1,2,3}^{pha}$ : AUC=0.777; $F_{2,3}^{pha}$ , AUC=0.776; $F_{1,2,4}^{pha}$ : ACU=0.762; $F_{2,3,4}^{CEV}$ : ACU=0.723; $F_{1,2,3,4}^{CEV}$ : ACU=0.718; and $F_{1,3}^{pha}$ : ACU=0.712) had better performance than $F_{3}^{pha}$ , which demonstrated that the employing of multiphasic features could achieve superior statistical performance for Fuhrman grade differentiation of ccRCC. *F*pha 1,2,3 showed its highest performance in all models (AUC=0.777, 95% CI: 0.732–0.836, accuracy=0.743, sensitivity=0.692, and specificity= 0.819). A significant difference was observed between $F_{1,2,3}^{pha}$ and $F_{3}^{pha}$ (*p* value=0.039), which demonstrates that the multi- phase-combined CECT radiomics models outperforms the radiomics model with single-phase radiomics features.

#### 3.2.2 Key feature analysis

**Figure 7:**
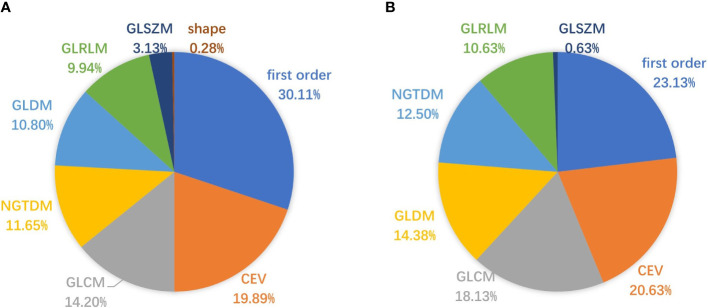
Pie charts representing the percentage (%) of key features selected by mRMR. **(A)** The percentage of key features in all radiomics models; **(B)** the percentage of key features in discriminative models.

**Figure 8:**
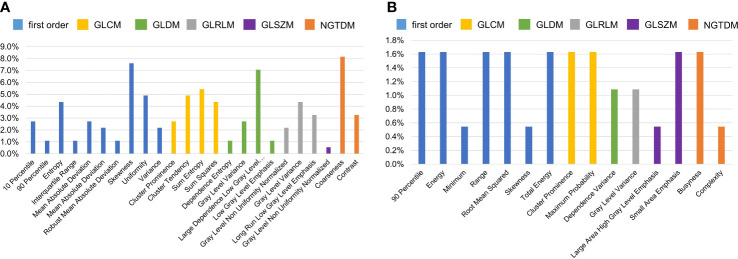
The frequency of key features selected by mRMR in discriminative models. The higher frequency of individual feature denoted that it played the more important role in the discriminative model. **(A)** The frequencies of key 3D classical radiomics features selected by mRMR in discriminative models; **(B)** the frequencies of CEV features selected by mRMR in discriminative models.

mRMR ranked all features and selected 20 key features to build radiomics models. We counted the frequency of these key features in all radiomics models, and their percentage is summarized in **Figures 7**, **8**. **Figure 7** summarizes the percentage of key features selected by mRMR in discriminative models. It showed that first-order, CEV, and GLCM types were the most frequently selected feature types in all models and discriminative models. There were 39 features selected by mRMR as key features (3D classical radiomics features=24, CEV feature=15) in discriminative models, and their frequencies are shown in **Figure 8**.

The top 10 most frequently selected features are shown in **Table 3**. In order to further verify the effectivity of the top 10 most frequently selected features, the significant differences in these features between low grade and high grade were calculated on the nephrographic phase, which is also summarized in **Table 3**. CEV features also played important roles in Fuhrman grading of ccRCC; the characterization of the most frequently selected CEV features is summarized in **Table 4**.

**Table 3:** The characterization of the top 10 most frequently selected features on nephrographic phase images.

Type	Name	Frequency	Low grade (mean ± SD)	High grade (mean ± SD)	*p-*value^a^
First-order	Skewness (2nd)	7.61%	2.83 ± 0.24	2.68 ± 0.32	0.044^*^
	Uniformity (5th)	4.89%	0.46 ± 0.64	0.50 ± 0.55	0.793
	Entropy (7th)	4.35%	0.18 ± 0.04	0.19 ± 0.05	0.141
GLCM	Sum entropy (4th)	5.43%	3.57 ± 0.27	3.43 ± 0.36	0.117
	Cluster tendency (5th)	4.89%	10.22 ± 3.97	8.49 ± 3.69	0.112
	Sum squares (7th)	4.35%	3.20 ± 1.130	2.62 ± 1.03	0.044^*^
GLDM	Large dependence low gray level emphasis (3rd)	7.07%	1.09 ± 0.880	1.32 ± 1.04	0.416
GLRLM	Gray level variance (7th)	4.35%	3.89 ± 1.20	3.18 ± 1.19	0.037^*^
	Long run low gray level emphasis (10th)	3.26%	0.04 ± 0.03	0.05 ± 0.04	0.583
NGTDM	Coarseness (1st)	8.15%	0.02 ± 0.01	0.00 ± 0.01	0.010^*^

* p-value <0.05 was considered as statistically significant difference.

a Independent t-test.

**Table 4:** The characterization of the most frequently selected CEV features.

Feature name	Low grade (mean ± SD)	High grade (mean ± SD)	*p-*value^a^
90 Percentile (*F*_c_–*F*_n_)	−0.09 ± 22.82	−6.95 ± 21.15	0.278
Energy (*F*_d_–*F*_n_)	1.67×10^8^ ± 7.60×10^9^	4.09×10^9^ ± 5.81×10^9^	0.044^*^
Range (*F*_d_–*F*_n_)	−272.10 ± 540.50	−244.42 ± 445.02	0.848
Root mean squared (*F*_d_–*F*_n_)	9.29 ± 16.87	2.16 ± 14.01	0.118
Total energy (*F*_c_–*F*_n_)	1.60×10^8^ ± 7.60×10^9^	4.09×10^9^ ± 5.81×10^9^	0.043^*^
Cluster prominence (*F*_c_–*F*_n_)	−608.43 ± 1396.13	−295.93 ± 378.83	0.349
Maximum probability (*F*_c_–*F*_n_)	0.01 ± 0.04	0.02 ± 0.04	0.559
Small area emphasis (*F*_c_–*F*_n_)	0.01 ± 0.02	0.01 ± 0.02	0.383
Busyness (*F*_d_–*F*_n_)	4.881 ± 14.612	15.994 ± 27.647	0.048^*^

* p-value <0.05 was considered as statistically significant difference.

a Independent t-test.

## 4 Discussion

The originality of this retrospective study was to proposed multi-phase-combined CECT radiomics models for the noninvasive Fuhrman grading of ccRCC, which concatenated multi-phase 3D radiomics features and CEV features to comprehensively reflect the microscale information of tumors.

Some studies have shown that single-phase radiomics model may be a promising noninvasive method to predict the Fuhrman grade of ccRCC (37, 38). Demirjian et al. (39) evaluated the effectiveness of CT-based radiomics features in discriminating Fuhrman grades and TNM stages. Saelin et al. (40) evaluated some statistical differences in sex, age, tumor size, and CT imaging features according to the Fuhrman grade of ccRCCs. They found intratumoral necrosis on CT was a strong and independent predictor of biologically aggressive ccRCCs. Hussain et al. (41) proposed a learnable image histogram in a deep neural network framework that can learn task-specific image histograms. This method learned a statistical context features directly from the images and deploys it to extract representative discriminant textural image features.

Lin et al. (24) built a machine learning model to predict Fuhrman grades by combining three phases of CECT and claimed that it achieved superior statistical performance compared with the single-phase radiomics model. Ding et al. (12) incorporated texture features and six non-texture features for preoperatively differentiating Fuhrman grades. The texture features were extracted from the corticomedullary- and nephrographic-phase CECT images. The LASSO (42) was used to select the most valuable texture features and calculate a texture score for each patient. A logistic regression model was used to discriminate the high- from low- grade ccRCC at nephrectomy. Mostafa et al. (43) used three-phase CT scans (unenhanced, corticomedullary phase, and nephrographic phase) to build radiomic models. They first applied Laplacian of Gaussian and wavelet filter on delineated tumor volumes and then extracted tumor shape, size, intensity statistics, and texture from each segmented tumor volume. They selected features to build three classification models (SVM, random forest, and logistic regression) to discriminate Fuhrman grades. Shu et al. (15) extracted 1,029 radiomic features from corticomedullary and nephrographic phases and then used LASSO regression method to select features. Then, the selected features were constructed using three classification models (corticomedullary phase, nephrographic phase, and their combination) by logistic regression method to discriminate high- and low- grade ccRCC. Feng et al. (44) also used three-phase CECT (non-contrast phase, corticomedullary phase, and nephrographic phase) to extract first-order image features and reflect tumor heterogeneity. They also found that entropy, which reflects texture irregularity and chaos, was an independent and excellent texture feature to discriminate Fuhrman grades. However, they did not comprehensively investigate the performance of different phases and their combinations.

In this study, we built multi-phase- combined radiomics models by extracting 3D classical radiomics features and computing CEV features from the entire 3D tumor mass. The following were the innovations of this study. (1) We extracted radiomics features from four-phase CECT to build multi-phase-combined radiomics models for ccRCC Fuhrman grading. The advantage of multi-phase-combined radiomics models is that the multi-phase features may be theoretically related to the hemodynamics and micro-vessel density of ccRCC tumors (45–47), since ccRCC is a highly angiogenic and vascularized tumor type (48). The experiments also showed that the combined model with the corticomedullary phase and the nephrographic phase could identify Fuhrman grades with the best performance. (2) We introduced 3D CEV features to quantitatively represent the variation in classical radiomics features between different phases, since the variation of first-order and texture features may reflect the enhancement of the inhomogeneity of the ccRCC tumor mass (49, 50). CEV features could serve as potential radiology biomarkers to macroscopically depict the hemodynamics and micro-vessel density of ccRCC tumors. (3) We comprehensively investigated the roles of classical radiomics features, CEV features, and their combinations, found that the first- order, CEV, and GLCM feature types played key roles in ccRCC Fuhrman grading.

Our study showed that there was one single-phase model ( $F_{3}^{pha}$ ) and seven multi-phase-combined models ( $F_{1,3}^{pha}$ , $F_{2,3}^{pha}$ , $F_{1,2,3}^{pha}$ , $F_{1,2,4}^{pha}$ , and $F_{1,2,3,4}^{pha}$ , $F_{2,3,4}^{CEV}$ , $F_{1,2,3,4}^{CEV}$ ) considered as discriminative model (AUC>0.7). The statistical results demonstrated that five multi-phase-combined radiomics models had better performance than single-phase models, of which $F_{2,3}^{pha}$ and $F_{1,2,3}^{pha}$ model shown the highest AUC values in all models. The performance of $F_{2,3}^{pha}$ and $F_{1,2,3}^{pha}$ is significantly better than $F_{3}^{pha}$ (*p*- value <0.05). Compared with other single-phase models, the $F_{3}^{pha}$ model showed its high performance, whose AUC value was comparable to some multi-phase-combined radiomics models. In addition, four discriminative multi-phase-combined radiomics models ( $F_{1,2,3}^{pha}$ , $F_{2,3}^{pha}$ , $F_{1,3}^{pha}$ , and $F_{1,2,3,4}^{pha}$ ) included nephrographic phase features, which denoted that nephrographic phase features played a key role in distinguishing Fuhrman grades of ccRCC.

First-order, CEV, and GLCM feature types were the top 3 most frequently selected feature types in discriminative models, meaning that they played crucial roles in Fuhrman grade prediction. The top 10 most frequently selected features respectively belong to first-order type (n=3), GLCM type (n=3), GLDM type (n=1), GLRLM type (n=2), and NGTDM type (n=1). Coarseness was the top 1 most frequently selected feature in discriminative models. It captures the spatial change rate of gray-level intensities and reflects the gray level difference between the central pixel and its neighborhoods. Skewness is the second most frequently selected feature, which measures the asymmetry of the gray-level intensity distribution curve. Large dependence, low gray-level emphasis is the third most frequently selected feature, which measures the joint distribution of large dependence with lower gray-level values. Entropy and sum entropy measure the inherent randomness of gray-level intensities, which reflect the tumor heterogeneity and were usually considered as biomarkers for tumor stage in radiation oncology (15, 44, 51).

Two models combining CEV features ( $F_{2,3,4}^{CEV}$ , $F_{1,2,3,4}^{CEV}$ ) were considered as discriminative models, which denoted that CEV features could be used as supplementary features for decision-making in Fuhrman grading. There were 15 CEV features ranked as important feature by mRMR, of which the variation in first-order features (frequency=9.24%) made up the highest proportion in the CEV feature group.

There were some limitations in this study. It was a relatively small sample size, and there was no external test set due to our strict inclusion criteria, which required all four phases of contrast-enhanced CT to be available for all patients. The radiomics applications would suffer from poor external validation due to inter-institutional variations of CT protocol and workflow. Therefore, a prospective and multicenter experimental study was necessary for further validation in an independent cohort. The second limitation is the use of the Fuhrman nuclear grading system instead of the latest WHO/ISUP grading system (52–54), as some of the included cases date back to 2009 when the Fuhrman grading system was widely used. The third limitation is that only 110 3D classical radiomics features were extracted from each phase to build the models. Some preprocessing methods (e.g., Laplacian of Gaussian, wavelet filter, gradient filter, and local binary pattern filter) could generate derived images, and more features extracted from derived images could enhance the performance of radiomic models (43). Moreover, the AUC value will be higher if clinical variables are incorporated into the models (55, 56).

In conclusion, the multi-phase-combined CECT radiomics methods could noninvasively predict the Fuhrman grades of ccRCC. The multi-phase features with 3D classical radiomics features and CEV features may serve as noninvasive radiology biomarkers for Fuhrman grade prediction.

## Data availability statement

The original contributions presented in the study are included in the article/**Supplementary Material**. Further inquiries can be directed to the corresponding author.

## Ethics statement

Institutional Review Board approval was not required because this is a retrospective study with anonymous data. Study subjects or cohorts have not been previously reported in other publications. Written informed consent was not required for this study because this is a retrospective study with anonymous data. 

## Author contributions

ZZ: Conceptualization, Methodology, Funding acquisition, Writing-Original Draft. XQ: Software, Validation. JH: Formal analysis. CG: Validation. YZ: Validation. XD: Investigation. TC: Formal analysis. JZ: Resources, Writing-Review & Editing, Funding acquisition. YD: Writing-Review & Editing, Funding acquisition. All authors contributed to the article and approved the submitted version.
