# Supplementary material for: Multi-phase-combined CECT radiomics models for Fuhrman grade prediction of clear cell renal cell carcinoma
Source: Front Oncol. 2023 Aug 23;13:1167328. doi: 10.3389/fonc.2023.1167328 (PMC10485140; doi:10.3389/fonc.2023.1167328)
Supplement: Supplementary file 1 [file Table_1.docx]

# Appendix A

Table S1: Feature categories and names

| Category | Feature Name |
| --- | --- |
| first order (n=19) | energy, total energy, entropy, minimum, 10th percentile,90th percentile, maximum, mean, median, interquartile range, range, mean absolute deviation, robust mean absolute deviation, root mean squared, standard deviation, skewness, kurtosis, variance, uniformity |
| shape (n=17) | mesh volume, voxel volume, surface area, surface area to volume ratio, sphericity, compactness 1, compactness 2, spherical disproportion, maximum 3d diameter, maximum 2d diameter (slice), maximum 2d diameter (column), maximum 2d diameter (row), major axis length, minor axis length, least axis length, elongation, flatness |
| GLCM  (n=24) | autocorrelation, joint average, cluster prominence, cluster shade, cluster tendency, contrast, correlation, difference average, difference entropy, difference variance, joint energy, joint entropy, informational measure of correlation 1, informational measure of correlation 2, inverse difference moment, maximal correlation coefficient, inverse difference moment normalized, inverse difference, inverse difference normalized, inverse variance, maximum probability, sum average, sum entropy, sum of squares |
| GLSZM (n=16) | small area emphasis, large area emphasis, gray level non-uniformity, gray level non-uniformity normalized, size-zone non-uniformity, size-zone non-uniformity normalized, zone percentage, gray level variance, zone variance, zone entropy, low gray level zone emphasis,  high gray level zone emphasis, small area low gray level emphasis, small area high gray level emphasis, large area low gray level emphasis, large area high gray level emphasis |
| GLDM (n=14) | small dependence emphasis, large dependence emphasis, gray level non-uniformity, dependence non-uniformity, dependence non-uniformity normalized, gray level variance, dependence variance, dependence entropy, low gray level emphasis, high gray level emphasis, small dependence low gray level emphasis, small dependence high gray level emphasis, large dependence low gray level emphasis, large dependence high gray level emphasis |
| GLRLM (n=16) | short run emphasis, long run emphasis, gray level non-uniformity, gray level non-uniformity normalized, run length non-uniformity, run length non-uniformity normalized, run percentage, gray level variance, run variance, run entropy, low gray level run emphasis, high gray level run emphasis, short run low gray level emphasis, short run high gray level emphasis, long run low gray level emphasis, long run high gray level emphasis |
| NGTDM (n=5) | coarseness, contrast, busyness, complexity, strength |
